# Spatial heterogeneity of hemorrhagic fever with renal syndrome is driven by environmental factors and rodent community composition

**DOI:** 10.1371/journal.pntd.0006881

**Published:** 2018-10-24

**Authors:** Hong Xiao, Xin Tong, Lidong Gao, Shixiong Hu, Hua Tan, Zheng Y. X. Huang, Guogang Zhang, Qiqi Yang, Xinyao Li, Ru Huang, Shilu Tong, Huaiyu Tian

**Affiliations:** 1 College of Resources and Environmental Sciences, Hunan Normal University, Changsha, Hunan Province, China; 2 Key Laboratory of Geospatial Big Data Mining and Application, Changsha, Hunan Province, China; 3 State Key Laboratory of Remote Sensing Science, College of Global Change and Earth System Science, Beijing Normal University, Beijing, China; 4 Hunan Provincial Center for Disease Control and Prevention, Changsha, Hunan Province, China; 5 School of Biomedical Informatics, The University of Texas Health Science Center at Houston, Houston, Texas, United States of America; 6 College of Life Sciences, Nanjing Normal University, Nanjing, Jiangsu Province, China; 7 Key Laboratory of Forest Protection of State Forestry Administration, National Bird Banding Center of China, Research Institute of Forest Ecology, Environment and Protection, Chinese Academy of Forestry, Beijing, China; 8 Shanghai Children’s Medical Center, Shanghai Jiao Tong University, Shanghai, China; 9 School of Public Health and Institute of Environment and Population Health, Anhui Medical University, Hefei, Anhui Province, China; 10 School of Public Health and Social Work, Queensland University of Technology, Kelvin Grove, Queensland, Australia; The University of Kansas, UNITED STATES

## Abstract

Hemorrhagic fever with renal syndrome (HFRS) is a rodent-borne disease caused mainly by two hantaviruses in China: Hantaan virus and Seoul virus. Environmental factors can significantly affect the risk of contracting hantavirus infections, primarily through their effects on rodent population dynamics and human-rodent contact. We aimed to clarify the environmental risk factors favoring rodent-to-human transmission to provide scientific evidence for developing effective HFRS prevention and control strategies. The 10-year (2006–2015) field surveillance data from the rodent hosts for hantavirus, the epidemiological and environmental data extracted from satellite images and meteorological stations, rodent-to-human transmission rates and impacts of the environment on rodent community composition were used to quantify the relationships among environmental factors, rodent species and HFRS occurrence. The study included 709 cases of HFRS. Rodent species in Chenzhou, a hantavirus hotspot, comprise mainly *Rattus norvegicus*, *Mus musculus*, *R*. *flavipectus* and some other species (*R*. *losea* and *Microtus fortis calamorum*). The rodent species played different roles across the various land types we examined, but all of them were associated with transmission risks. Some species were associated with HFRS occurrence risk in forest and water bodies. *R*. *norvegicus* and *R*. *flavipectus* were associated with risk of HFRS incidence in grassland, whereas *M*. *musculus* and *R*. *flavipectus* were associated with this risk in built-on land. The rodent community composition was also associated with environmental variability. The predictive risk models based on these significant factors were validated by a good-fit model, where: cultivated land was predicted to represent the highest risk for HFRS incidence, which accords with the statistics for HFRS cases in 2014–2015. The spatial heterogeneity of HFRS disease may be influenced by rodent community composition, which is associated with local environmental conditions. Therefore, future work should focus on preventing HFRS is moist, warm environments.

## Author summary

Hantaviruses are responsible for a disease called hemorrhagic fever with renal syndrome (HFRS), which is characterized by fever, headache, back pain, abdominal pain, hypotension, multi-systemic hemorrhage and acute renal failure in humans. China has the highest incidence of HFRS worldwide, accounting for 90% of global cases reported. Many factors can influence the incidence of HFRS, but the two main ones are related to the composition of the rodent community composition and the influence of the environment in the transmission of this disease. Here, we used a combination of 10-year (2006–2015) field surveillance on rodent reservoir of HFRS along with epidemiological and environmental data to investigate the factors related to the transmission of HFRS infections. Our results indicate that the occurrence of HFRS is influenced by the potential for rodent-to-human contact under different environmental conditions. Our findings, which offer novel insights into the networks used by hantavirus for transmission, could be used to optimize preventative interventions against HFRS disease.

## Introduction

Hemorrhagic fever with renal syndrome (HFRS) is a rodent-borne infectious disease caused mainly by two hantaviruses in China: Hantaan virus (HTNV) and Seoul virus (SEOV) [1]. In humans, HFRS is clinically characterized by fever, hemorrhage (vascular leakage resulting in hemorrhagic manifestations), headache, back pain, abdominal pain, and acute kidney damage [2,3]. HFRS cases have occurred mainly in China, the Republic of Korea, and the Far East region of the Russian Federation in Asia, as well as in Finland, Sweden and western and central Europe [4]. With the highest occurrence of HFRS, China has experienced approximately 90% of the cases worldwide over the last few decades [5,6]. From 2006 to 2015, more than 110,000 cases of HFRS were reported in China [7], making HFRS a critical public health issue in this country [8].

Hunan Province, which is located in the middle reaches of the Yangtze River in central China, is one of the areas in this country where HFRS is most highly endemic [9,10], with 6,237 cases of HFRS diagnosed during 2006–2015. Chenzhou, a city with a subtropical climate in Hunan Province, is a typical hotspot for hantavirus infections. SEOV and HTNV have both been reported in Hunan Province, with SEOV being the dominant strain [11,12]. The main hosts for SEOV are *Rattus norvegicus* [13], *Rattus flavipectus*, and *Mus musculus* [14], whereas *Apodemus agrarius* is the host for HTNV [15,16].

Transmission of hantavirus to humans occurs via inhalation of aerosolized viral particles present in the urine, feces, and saliva excreted into the environment by rodents infected with it [17,18]. Consequently, rodent-to-human hantavirus transmission depends on the following factors: 1) the frequency of contact between human and rodent populations, which is associated with human activities, living conditions, working conditions, host species distributions, rodent population densities, and virus prevalence in rodents [19–21], and 2) external environmental factors including temperature, rainfall, relative humidity, land type, vegetation status, soil moisture status, and elevation, which play important roles in reservoir host density and the level of exposure to infectious viruses [22–26]. Previous studies have shown the tendency for rodent species to thrive in restricted habitats. *A*. *agrarius*, for example, prefers humid and food-rich environments and is abundant in forested regions and fields. In contrast, *R*. *norvegicus* is found predominantly in residential areas and is the main vector for zoonotic diseases in rural and urban populations [27,28]. Environmental factors can influence the occurrence of HFRS through their effects on the reservoir hosts and their living conditions [29–32]. However, the specific relationships among different environmental factors, different rodent species, and the occurrence of HFRS remain unclear.

The purpose of this study was to determine how the rodent community composition influences the risk of contracting a human hantavirus infection in different environments. Various techniques for forecasting environmental changes, such as the ability to forecast trends in climate change, have advanced significantly. Therefore, constructing reliable models with which to quantify the relationships among the environments, rodent populations, and the occurrences of human HFRS is necessary to predict the risk of HFRS occurrence to help to prevent and control HFRS epidemics.

## Methods

### Ethics statement

The study was approved by the Research Institutional Review Board of the Hunan Provincial Centre for Disease Control and Prevention (CDC). In this study, all the medical data from the patients were anonymized to ensure confidentiality. Only aggregated data were used in the data analysis, and no personal information was utilized. Hunan CDC gave the approval for the trapping and investigation of rodents. All the procedures carried out on rodents were performed in accordance with the animal welfare legislation for the protection of animals used for scientific purposes in China. The research protocol’s ethical terms (HFRS HN-01 research program, No. HN2008001) were approved by the Animal Ethics Committee of the Hunan CDC.

### Geographic profile of the study area

**Fig 1:**
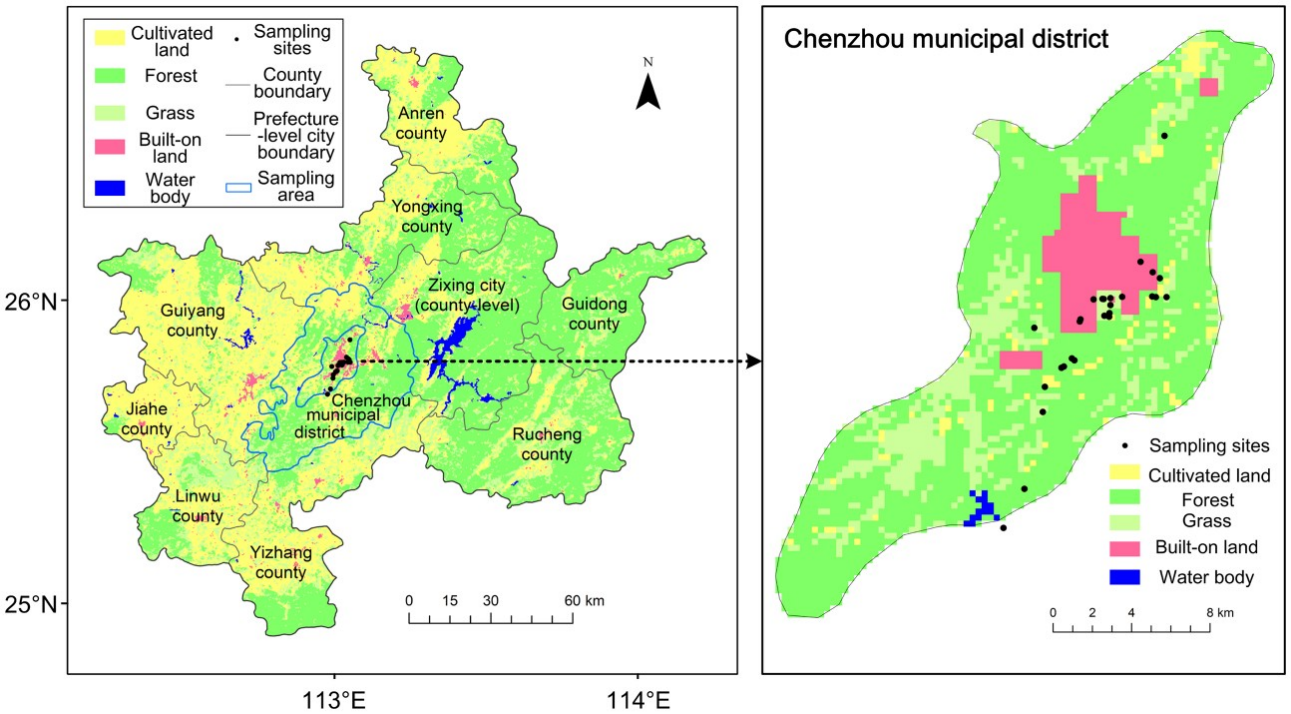
Land uses of the study area of Chenzhou City (prefecture-level) in 2015, and the sampling sites locations used for rodent surveillance. Different colors in the base map represent the different types of land use (yellow: cultivated land; green: forest; light green: grass; rose: built-on land; dark blue: water body). The dark points and areas within the blue line show the locations of the sampling sites and areas of rodent surveillance, respectively. The gray and dark lines are boundaries at county and prefecture-level city, respectively. The maps for the land use types in Chenzhou City during 2006–2015 can be found in the appendix.

Chenzhou (112°13′-114°14′E, 24°53′-26°50′N) is located in the southeast region of Hunan Province in Central China. The region is 217 km wide and 202 km long, with a total land area of 19,400 km^2^. Chenzhou includes two municipal districts (Beihu and Suxian) and nine counties (Guiyang, Yizhang, Yongxing, Jiahe, Linwu, Rucheng, Guidong, Anren, and Zixing) with a population of about 4.7 million [33]. Chenzhou has a humid subtropical climate and an annual average temperature of 19°C. It is covered mainly by mountains and hills, with similar amounts of hillocks and plains. The terrain is high in the southeast, low in the northwest, with the southeast being mountainous, and the northwest mainly having hills, hillock, and plain (Fig 1).

### Data collection

The data for the HFRS cases that occurred in Chenzhou (January, 2006 to December, 2015) were obtained from the Hunan CDC. All cases were initially diagnosed according to the clinical criteria of the Ministry of Health of China. The serum samples collected from patients with clinical signs of HFRS were sent to the Hunan CDC laboratory. The diagnosis was supported by detection of specific IgM and IgG antibodies to hantavirus in acute-phase serum specimens by enzyme-linked immunosorbent assay. In this study, HTNV and SEOV infections were not distinguished from each other. All cases were geo-coded according to the residential address using Google Earth (Google, Mountain View, CA, USA) and classified based on which type of land was nearest to the address.

Annual land data from 2006 to 2015 were extracted from the Climate Change Initiative Land Cover Project, with a spatial resolution of 300 m. The study areas were classified into five land types: cultivated land, forest, grassland, building land, and water bodies (rivers, lakes, etc.). Spatial data were analyzed in ArcGIS 10.1 (Esri Inc., Redlands, CA, USA).

From January, 2006 to December, 2015, meteorological data (daily precipitation, daily average temperature, and daily average relative humidity) were obtained from the China Meteorological Data Sharing Service System (http://cdc.cma.gov.cn/index.jsp). The data were integrated into monthly average values so as to prepare for the next calculation. To characterize the vegetation and soil moisture status, the normalized difference vegetation index (NDVI) and the temperature vegetation dryness index (TVDI), were extracted from multi-day composited Moderate-Resolution Imaging Spectroradiometer (MODIS) data from January 2006 to December 2015. The MODIS data (MOD11A2 and MOD13A2) were acquired with a spatial resolution of 1 km from the Land Processes Distributed Active Archive Center (LP DAAC/NASA, https://lpdaac.usgs.gov). The NDVI is an index relating to the amount and productivity of vegetation and is always used as an important environmental parameter in studies of epidemics [34,35]. The downloaded remote sensing data for NDVI was 16-day composited, whereas data for land surface temperature (LST) was 8-day composited. We composited both of them into monthly data, cut the extent of Chenzhou City out, and calculated the mean NDVI value for Chenzhou City monthly. After that, the monthly NDVI and LST data were used to calculate the TVDI through a special tool of ENVI 5.0 (Esri Inc., Redlands, CA, USA). The TVDI, which ranges from 0 to 1, is used extensively in soil moisture estimation. A higher TVDI corresponds to lower soil moisture and vice versa. The TVDI is estimated using the following equation:

$$TVDI=\frac{T_{s}-T_{s\text{min}}}{a+bNDVI-T_{s\text{min}}}$$ (1)

where *T*_*s*_ is the observed land surface temperature at a given pixel; *T*_*smin*_ is the minimum land surface temperature for a given NDVI, defining the wet edge; and *a* and *b* are the parameters defining the dry edge. The data are fitted to a linear model (*T*_*smax*_ = *a + b*NDVI, detailed values of *a* and *b* were in the appendix support information).

Environmental matrices consist of the annual variances of the environmental variables. For each type of environmental variables, the monthly average values were obtained and used to calculate the standard deviations and mean values for each year. The coefficient of variances for each year were estimated and then arranged into an array (i.e. an environmental matrix *H*).

### Field surveillance

From 2006 to 2015, the surveillance of rodent hosts in Chenzhou was conducted once per month over three consecutive nights. In total, 28 permanent trapping sites were established among the following three distinct environments: 14 in special industrial areas (restaurants and processing plants), 8 in residential areas, and 6 in rural areas. From 2006 to 2015, 72,039 effective trap-nights were set. At least 300 medium-sized peanut-baited steel traps were set each night and recovered in the morning for all the trapping sites. More than 100 traps were placed indoors and more than 200 traps were placed outdoors. The outdoors traps were set along the terrain at intervals of 10 m with the column spacing set at 50 m, and indoors trap interval was approximately 12 to 15 meters. The trapped rodents were coded, and their species and sex were identified [36].

### Statistical analysis

**Fig 2:**
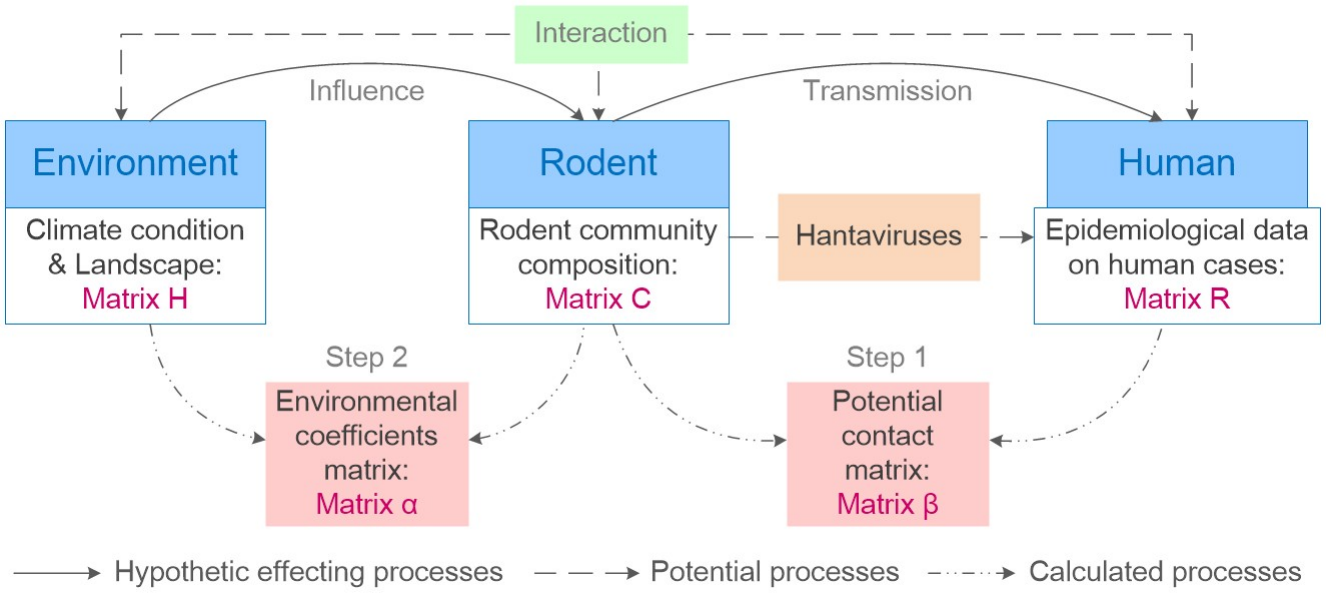
Hypothetical processes affecting the environment, rodents, and humans, and the matrix calculations. Words shown in blue are the monitored objects and pink boxes are the main coefficients that we calculated. All the matrices are named and emphasized in plum color. Green boxes represent potential interaction processes and orange boxes represent the viruses that couldn’t surveyed directly.

We created a potential contact matrix (*β*) and an environmental coefficient matrix (*α*) to quantify the associations among the environmental variables, rodent community composition, and the occurrence of HFRS in the two main linear fitting steps (Fig 2).

The first step was intended to clarify the influence of the rodent community composition on the occurrence of HFRS. Matrix *β* represented an influencing parameter that, can be calculated from matrices *R* and *C*. In detail, *R* represents a matrix comprising the proportions of HFRS cases among the different land-use types in different years, whose rows represent the proportions of HFRS cases for one land-use type in different years and whose columns represent the proportions of HFRS cases in the same year for the different land types. Matrix *C* was built using the rodent surveillance data. We calculated the proportion of different rodent species (*R*. *norvegicus*, *M*. *musculus*, *R*. *flavipectus*, and other rodent species, mainly *R*. *losea* and *Microtus fortis calamorum*) each year and arrange them as matrix *C*, with the rows representing the proportions of the same rodent species in different years and the columns representing the proportions of different rodent species in the same year. We then used the following formula to calculate matrix *β* so as to determine how the rodent community composition influenced the occurrence of HFRS in the different land-use types:

$$R_{ij}=\sum_{k=1}^{K}\beta_{ik}\times C_{kj}$$ (2)

where *R*_*ij*_ is the proportion of HFRS cases in area *i* in year *j*, *β*_*ik*_ is the potential contact probability between human and rodent species *k* in area *i*, and *C*_*kj*_ is the proportion of rodent species *k* in year *j* (*i* = 1,2,…,5; *j* = 1,2,…,10; *k* = 4).

To show the inner relationship for each matrix, formula (2) can also be written as:

$$\left(\begin{array}{cccc} R_{11} & R_{12} & \cdots & R_{1j}\\ R_{21} & R_{22} & \cdots & R_{2j}\\ \vdots & \vdots & \ddots & \vdots \\ R_{i1} & R_{i2} & \cdots & R_{ij} \end{array}\right)=\left(\begin{array}{cccc} \beta_{11} & \beta_{12} & \cdots & \beta_{1k}\\ \beta_{21} & \beta_{22} & \cdots & \beta_{2k}\\ \vdots & \vdots & \ddots & \vdots \\ \beta_{i1} & \beta_{i2} & \cdots & \beta_{ik} \end{array}\right)\cdot \left(\begin{array}{cccc} C_{11} & C_{12} & \cdots & C_{1j}\\ C_{21} & C_{22} & \cdots & C_{2j}\\ \vdots & \vdots & \ddots & \vdots \\ C_{k1} & C_{k2} & \cdots & C_{kj} \end{array}\right)$$ (3)

The second step involved evaluating the effects of the environmental variables on the rodents; matrix *α* was the influencing parameter. We used the environmental coefficients matrix *H* to simplify the assessment of the environmental influences. Environmental matrix *H* was constructed with rows representing the same environmental variable in different years and columns representing the proportions of different environmental variables (precipitation, temperature, relative humidity, NDVI, and TVDI) in the same year. The matrix *C*_*kj*_ represent the proportion of rodent species between years, the same as in Eqs (2) and (3). The environmental coefficient matrix *α* was calculated based on *C* and *H* to determine how the environmental variables influenced the rodent population dynamics:

$${\text{C}}_{kj}=\sum_{n=1}^{N}\alpha_{kn}\times H_{nj}$$ (4)

where *C*_*kj*_ is the proportion of rodent species *k* in year *j*, *H*_*nj*_ is the coefficient of variance (estimated from monthly values) of environmental variable *n* in year *j*, and *α*_*kn*_ is the influence of the environment variable *n* on rodent species *k* (*i* = 1,2,…,5; *j* = 1,2,…,10; *n* = 5).

Similarly, formula (4) can be written out in detail as well:

$$\left(\begin{array}{cccc} C_{11} & C_{12} & \cdots & C_{1j}\\ C_{21} & C_{22} & \cdots & C_{2j}\\ \vdots & \vdots & \ddots & \vdots \\ C_{k1} & C_{k2} & \cdots & C_{kj} \end{array}\right)=\left(\begin{array}{cccc} \alpha_{11} & \alpha_{12} & \cdots & \alpha_{1k}\\ \alpha_{21} & \alpha_{22} & \cdots & \alpha_{2k}\\ \vdots & \vdots & \ddots & \vdots \\ \alpha_{k1} & \alpha_{k2} & \cdots & \alpha_{kn} \end{array}\right)\cdot \left(\begin{array}{cccc} H_{11} & H_{12} & \cdots & H_{1j}\\ H_{21} & H_{22} & \cdots & H_{2j}\\ \vdots & \vdots & \ddots & \vdots \\ H_{n1} & H_{n2} & \cdots & H_{nj} \end{array}\right)$$ (5)

Based on the steps described above, we initially divided our data into two parts, where: 80% of the data were used to construct the matrices *R*, *C* and *H* (denoted as *R*_*0*_, *C*_*0*_ and *H*_*0*_, respectively), so as to calculate the parameter matrices *α* and *β* (denoted as *α*_*0*_ and *β*_*0*_, respectively), and the remaining 20% was used to test the model (i.e., the 2006–2013 data were used as the training data, while the 2014–2015 data were used as the test data). In detail, this 20% of the data was also constructed as matrices *R* and *H* (denoted as *R*_*00*_ and *H*_*0t*_, respectively) and then *H*_*0t*_ was substituted into formula (4) to get a testing matrix *C* (*C*_*0t*_). Next, *C*_*0t*_ was substituted into formula (2) to gain the newly matrix *R* (*R*_*0t*_). Thus, we tested our model by comparing *R*_*00*_ and *R*_*0t*_ (Pearson correlation analysis and R^2^ calculation).

To further validate our method, we divided the annual HFRS cases data into quadrants, above and below the median longitude and median latitude, which enabled us to develop a cross validation by setting the on-diagonal and off-diagonal data, respectively, in 2006–2013 as the training data [37]. Specifically, the on-diagonal HFRS data for 2006–2013 was used to construct the *R* (*R*_*1*_) matrix and the off-diagonal HFRS data for the same years was used to construct *R*_*2*_. Similarly, the on-diagonal HFRS data for 2014–2015 was used to construct the *R* (*R*_*10*_) matrix and the off-diagonal HFRS data for the same years was used to construct *R*_*20*_. We calculated two parameter matrices; namely, *β*_*1*_ and *β*_*2*_ according to formula (3) using (*R*_*1*_, *C*_*0*_) and (*R*_*2*_, *C*_*0*_), respectively. We then defined the matrix-containing proportion for the rodent species in 2014–2015 as matrix *C*_*t*_ and substituted it into formula (3) with *β*_*1*_ and *β*_*2*_ to obtain two new matrices (*R*_*1t*_ and *R*_*2t*_), respectively. Finally, we tested the correlation coefficients of the values from *R*_*1t*_ and *R*_*2*_ (and *R*_*2t*_ and *R*_*1*_) in the corresponding year using Pearson correlation analysis and by R^2^ value calculation.

## Results

### Occurrence of HFRS and rodent community composition

**Fig 3:**
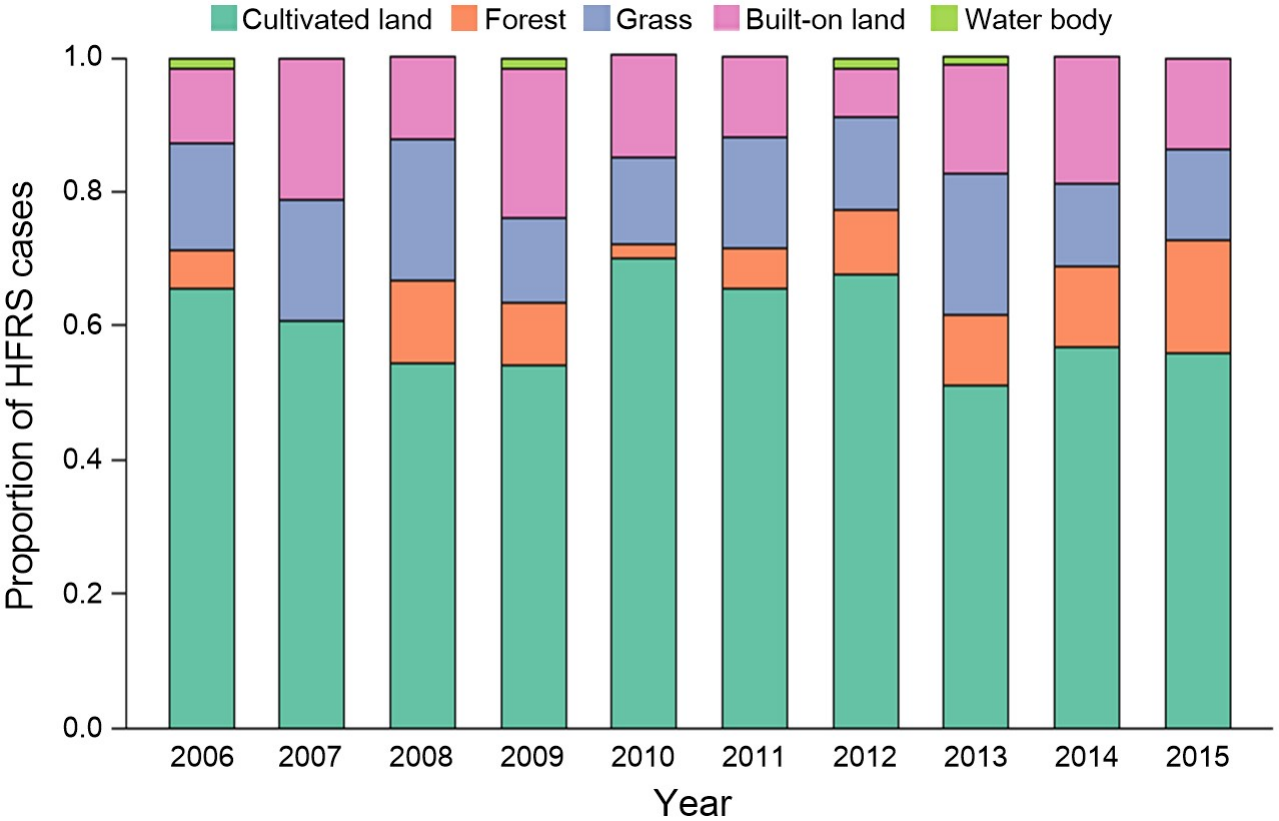
Annual proportion of HFRS cases among different land types in 2006–2015. Each column indicates the proportion of HFRS cases among the different land-use types (deep green: cultivated land; orange: forest; purple: grass; rose: built-on land; light green: water body) in a year.

In Chenzhou, 723 cases of HFRS were reported 2006–2015, of which 14 addresses (0.02%) could not be geocoded. The proportion of cases in association with each land type varied throughout the study period. Specifically, the highest proportion (> 50 percent) of affected patients lived near cultivated land, followed by built-on land and grassland with similar proportions. Relatively few cases of HFRS occurred in areas of forest lands. Furthermore, very few cases were reported in water-covered areas during the study period (Fig 3).

**Fig 4:**
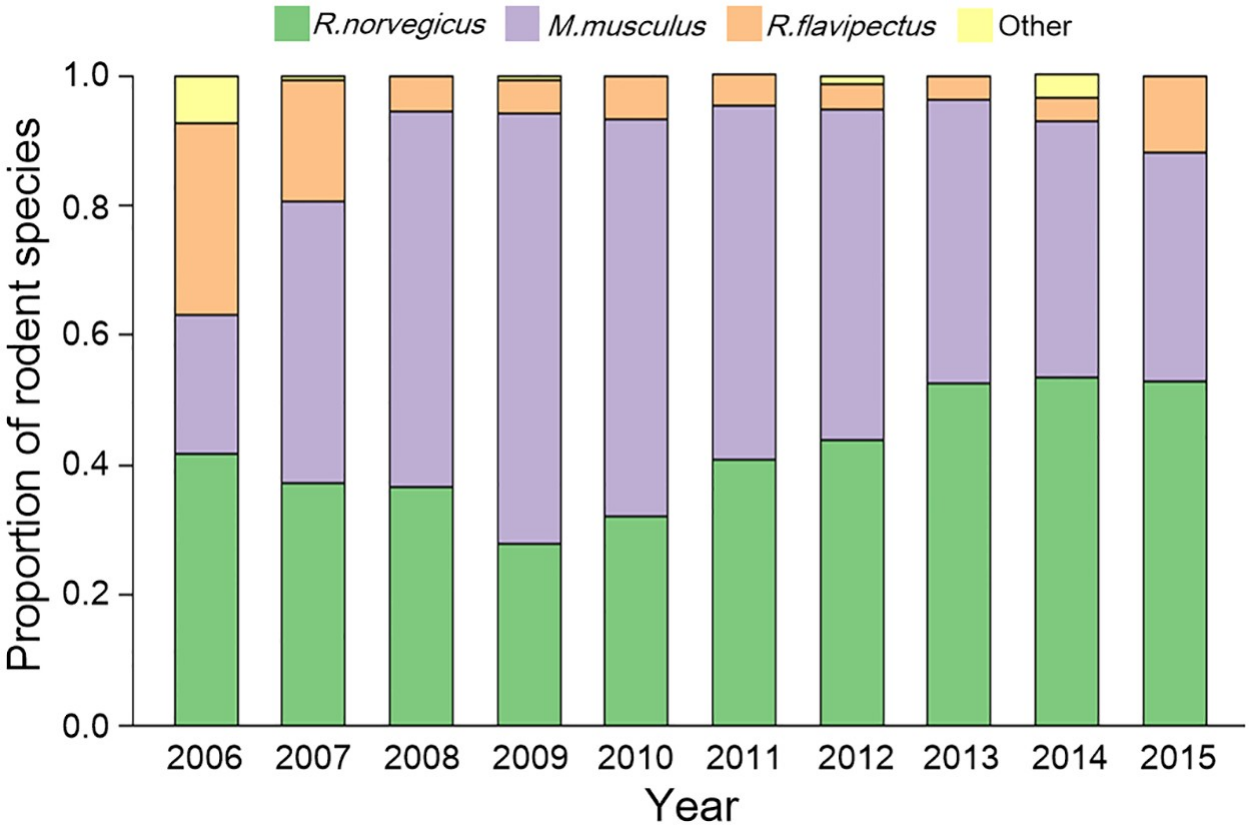
Proportion of each rodent species trapped in Chenzhou City, 2006–2015. Each column indicates the proportion of different rodent species (green: *R*. *norvegicus*; purple: *M*. *musculus*; orange: *R*. *flavipectus*; yellow: other species) in a year.

In total, 72093 traps were effective and 944 rodents were trapped, with *M*. *musculus* (38.77%) and *R*. *norvegicus* (48.20%) being the predominant species. The number of captured *R*. *flavipectus* and other species gradually decreased over time. The number of *M*. *musculus* increased each year from 2006 to 2009; however, after the peak (n = 93) in 2009, the number decreased. The number of *R*. *norvegicus* captured decreased from 68 to 34 rodents per year during 2006 to 2010, but 35 rodents were captured in 2011, after which downwards trend continued over the study period (Table 1). With respect to the rodent community composition per year (excluding 2006 and 2007), *M*. *musculus* and *R*. *norvegicus* comprised most of the trapped rodents (Fig 4).

**Table 1:** Number of rodents of each species trapped in Chenzhou, 2006–2015.

	*R*. *norvegicus*	*M*. *musculus*	*R*. *flavipectus*	Other species*
2006	68	35	48	12
2007	48	56	24	1
2008	47	74	7	0
2009	39	93	7	1
2010	34	65	7	0
2011	35	47	4	0
2012	33	38	3	1
2013	29	24	2	0
2014	15	11	1	1
2015	18	12	4	0

*Other species: *R*. *losea* and *Microtus fortis calamorum*

### Environmental factors

**Fig 5:**
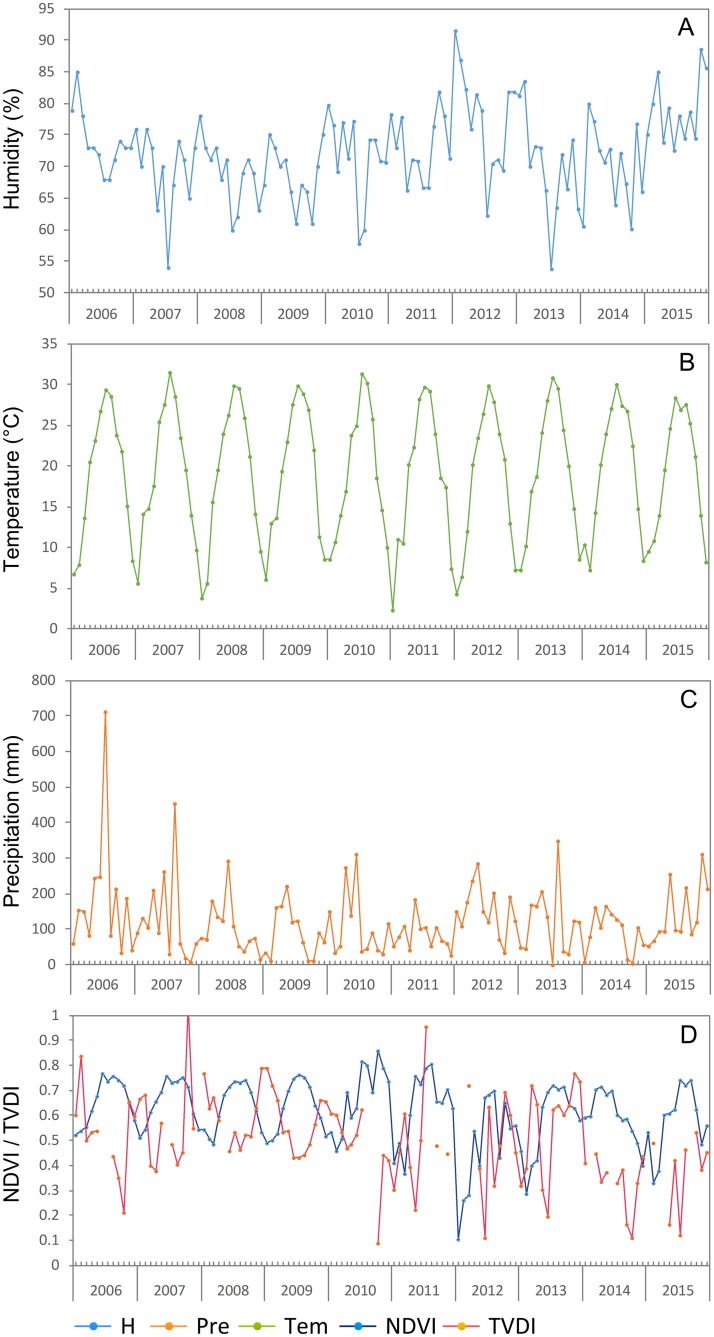
Monthly environmental variables data for Chenzhou, 2006–2015. The x-axis is arranged by year and within the segment of each year the values are ranked by month from January to December. Tem: temperature (°C), H: relative humidity (%), Pre: precipitation (mm), NDVI: the normalized difference vegetation index, TVDI: the temperature vegetation dryness index (the values in some moths were abnormal and have been removed from the analysis).

During the study period, the temperature and relative humidity maintained a certain degree of balance, while the precipitation level, NDVI, and TVDI all showed distinct and continuous fluctuation among the months and years (Fig 5). The temperature during the 10-year period maintained a similar variety among the months from 2°C to 33°C (Fig 5B) and the relative humidity remained at 70% to 80% (Fig 5A), but none of these parameters showed substantial interannual fluctuations. However, the precipitation levels fluctuated continuously over the 10-year period (Fig 5C). Additionally, although the NDVI and TVDI (the TVDI values in some months were abnormal and have been removed from the analysis) showed obvious fluctuations (Fig 5D), the variation coefficient for them for each year did not show a large difference.

### Associations among HFRS occurrences, rodents and environmental variables

**Fig 6:**
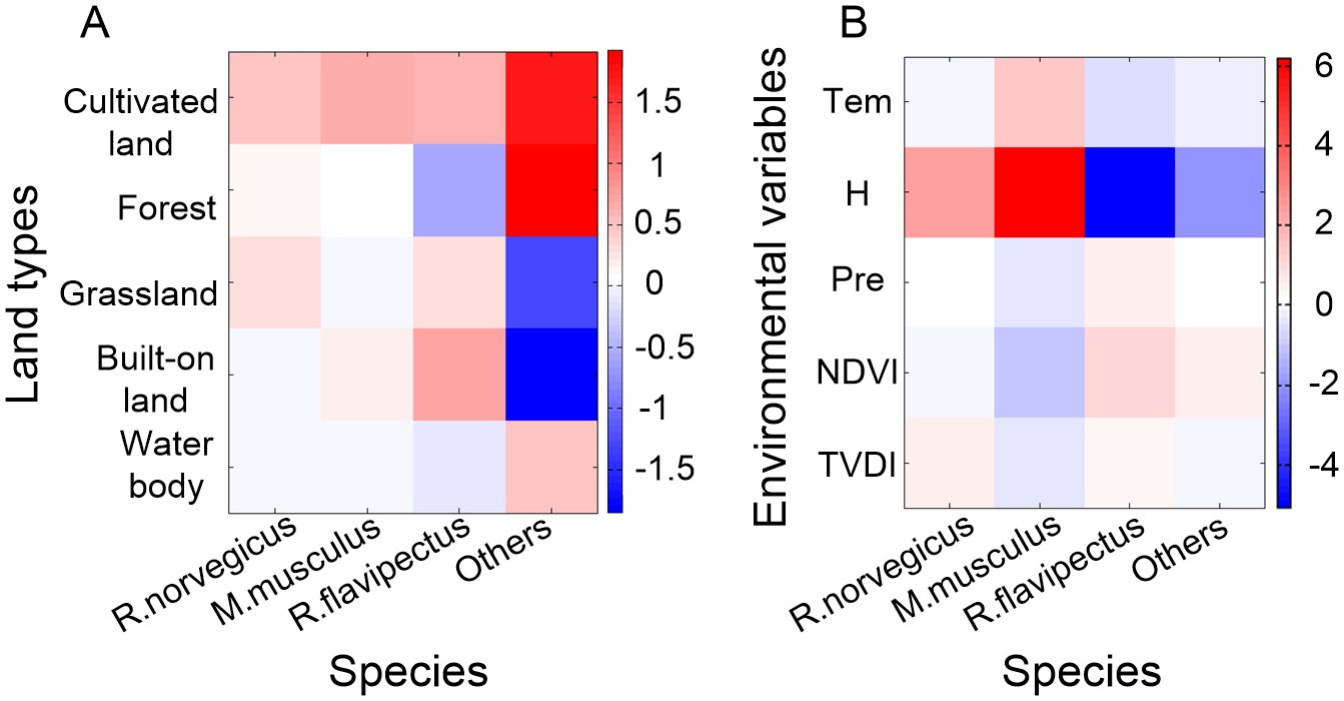
(A) Potential contact rates, as determined by potential contact matrix *β*, between humans and rodents among the different land-use types in Chenzhou; (B) Environment-to-rodent influence rates on different rodent species in Chenzhou for the different environmental variables, as determined by the environmental coefficient matrix *α*. Low values are shown in dark blue and high values in red. Positive values represent positive associations, and vice versa. Tem: temperature, H: relative humidity, Pre: precipitation, NDVI: the normalized difference vegetation index, TVDI: the temperature vegetation dryness index.

The potential contact *β* matrix and the environmental coefficient *α* matrix were visualized as shown in Fig 6, with low values colored in dark blue and high values in red. To unify the abscissas, the *α* matrix was transposed beforehand.

The potential contact *β* matrix shows the relationships among the different land types, various rodent species, and HFRS occurrences (Fig 5A). The main risk of HFRS occurrence on cultivated land was from all types of rodents in these areas. The main risk of HFRS occurrence in the forest was from other species (*R*. *losea* and *Microtus fortis calamorum*). Cases of HFRS in grassland areas resulted mainly from the presence of *R*. *norvegicus*, with *R*. *flavipectus* being slightly involved. The main risk on built-on land was from *R*. *flavipectus*. The other species (*R*. *losea* and *Microtus fortis calamorum*) were associated with the occurrence of HFRS in water-covered areas. We also noticed several negative values. Specifically, when species other than *R*. *norvegicus*, *M*. *musculus* and *R*. *flavipectus* accounted for a relatively large proportion of the rodent population, the occurrence of HFRS on grassland and built-on lands tended to decline. Similarly, when the proportion of *R*. *flavipectus* was large, the occurrence of HFRS in forest areas was low.

The environmental coefficient *α* matrix shows the influences of different environmental factors on the rodent community composition (Fig 6B). All rodent species were affected by relative humidity, while other factors influenced only some of the rodent species. There were positive, significant associations between relative humidity/TVDI and *R*. *norvegicus*, temperature/relative humidity and *M*. *musculus*, precipitation/NDVI and *R*. *flavipectus*, and NDVI/TVDI and other rodent species. Moreover, negative associations were seen with *R*. *norvegicus* for NDVI, with *M*. *musculus* for precipitation NDVI and TVDI, with *R*. *flavipectus* for temperature and humidity, and with humidity for the other species.

### HFRS risk

The results of the predicted probabilities of HFRS occurrence showed that cultivated land had the highest occurrence of HFRS among all land types in both 2014 and 2015. Grassland and built-on land showed a moderate or low risk of HFRS, and forest and water bodies had a low risk (Table 2). The Pearson correlation analysis revealed that *R*_*0t*_ was closely correlated with *R*_*00*_: r = 0.96 (P<0.01, n = 10) and R^2^ = 0.93. Thus, our prediction was able to reliably reflect the proportion of HFRS occurrences among the different land-use types.

**Table 2:** Predicted proportions and relative possibility of HFRS incidence risk and the number of observed HFRS cases among the different land types in 2014 and 2015.

	2014			2015		
	Proportion among all land-use types	Relative possibility of the risk	Observed HFRS cases	Proportion among all land-use types	Relative possibility of the risk	Observed HFRS cases
Cultivated land	0.50	1	33	0.54	1	33
Forest	0.04	0.09	7	0.06	0.09	10
Grassland	0.20	0.41	7	0.13	0.23	8
Built-on land	0.18	0.37	11	0.10	0.16	8
Water body	0	0	0	0.01	0	0

In addition, the prediction using off-diagonal data to predict the HFRS occurrence proportions among the different land-use types in the on-diagonal area (Table 3) showed an improved effect: r = 0.94 (P < 0.01, n = 10) and R^2^ = 0.88. In contrast, the prediction of HFRS occurrence proportions among the different land-use types in the off-diagonal area (Table 4) performed less well: r = 0.76 (P < 0.05, n = 10) and R^2^ = 0.58, and should therefore be regarded as having only slight credibility. Such cross-validation proved the reliability of the formula (2) again.

**Table 3:** Predicted proportions and relative possibility of HFRS incidence risk and the number of observed HFRS cases among the different land-use types in 2014 and 2015. (On-diagonal data to predict the off-diagonal risk).

	2014			2015		
	Proportion among all land-use types	Relative possibility of the risk	Observed HFRS cases	Proportion among all land-use types	Relative possibility of the risk	Observed HFRS cases
Cultivated land	0.51	1	18	0.58	1	15
Forest	0.18	0.32	3	0.04	0.06	9
Grassland	0.19	0.34	7	0.17	0.30	5
Built-on land	0.08	0.11	10	0.21	0.36	7
Water body	0.03	0	0	0	0	0

**Table 4:** Predicted proportions and relative possibility of HFRS incidence risk and the number of observed HFRS cases among the different land-use types in 2014 and 2015. (Off-diagonal data to predict the on-diagonal risk).

	2014			2015		
	Proportion among all land-use types	Relative possibility of the risk	Observed HFRS cases	Proportion among all land-use types	Relative possibility of the risk	Observed HFRS cases
Cultivated land	0.83	1	15	0.56	1	18
Forest	0.14	0.16	4	0.06	0.11	1
Grassland	0.05	0.06	0	0.32	0.57	3
Built-on land	0	0	1	0.05	0.08	1
Water body	0.03	0.03	0	0	0	0

## Discussion

The environment and host population are important factors affecting the dynamics of the natural foci of many diseases. For example, a less hot and humid climate may promote the prevalence of diseases transmitted by mosquitoes and other vectors, such as dengue [38], plague [39], and Lyme disease [40], among many others. The growth of rodent communities may increase the risk of hantavirus pulmonary syndrome [41]. We argue that clarification of the associations among the environment, host population, and disease dynamics is of great importance to the prevention and control of many types of disease epidemics. In this study, we quantified the relationships among the occurrence of HFRS, the environmental conditions, and the rodent species composition.

We found that the HFRS cases occurred predominantly in areas where the land has been cultivated, by grassland areas, built-on land, and forest. Very few cases were identified near water-covered areas in Chenzhou. This indicates that water bodies are relatively safe compared with the other land types we examined. The spread of hantavirus within different land types might be indirectly influenced by different environmental factors. In cultivated land, forest, grassland built-on land and water bodies, hantavirus transmission was influenced mainly by temperature, humidity precipitation, vegetation and soil moisture status; vegetation status; humidity, precipitation, vegetation and soil moisture status; temperature, humidity, precipitation and vegetation status; and soil moisture status, respectively.

Most HFRS cases were associated with cultivated land, possibly because it provides an environment with adequate food and an ideal habitat for rodent survival. Moreover, this type of land is closely linked with the presence of human beings. The rodent community composition was most strongly affected by relative humidity and the NDVI, followed by the TVDI, temperature, and precipitation. The TVDI is a complex variable that reflects the moisture condition, temperature, and vegetation lushness [42]. We hypothesize that the TVDI is a useful indicator of food availability for rodents and is directly influenced by climatic forcing. The NDVI reflects the level of vegetation coverage [43], is an accurate indicator of food and living conditions for rodents, and is related to the amount and productivity of vegetation and crops [44]. Most rodent species respond directly to fluctuations in food availability, and rodent species densities are driven by changes in food resources. Vegetation also provides shelter and safety from predators and other threats. Appropriate levels of precipitation and humidity not only provide ideal conditions for the plentiful growth of crops and help to boost rodent populations, but also influence rodent activity and thus affect hantavirus infectivity. Farmers working on cultivated land have more opportunities than humans elsewhere to come into contact with rodents, which increases of HFRS transmission risk [45,46]. Additionally, in the present study, more HFRS cases were distributed in grassland regions, and the main rodent species in this type of land were *R*. *norvegicus* and *R*. *flavipectus*. Relatively warmer temperatures provide a suitable environment for gestation and sexual maturation, and *M*. *musculus*, *R*. *norvegicus* and *M*. *musculus* might prefer humid environments, so when the relative humidity increases, the occurrence of HFRS in forest and grassland regions would rise. We found built-on land posed an important risk for HFRS occurrence, and the predominant rodent species found there were *R*. *flavipectus* and *M*. *musculus*. The risks from this land-use type might be related to urbanization and migrant workers. What should not be ignored is the fact that rodent abundances can be influenced by some specific activities, such as continuous pest control. Whether these activities have variable effects on the different rodent species needs to be investigated in future.

In our comparison of the observed versus the predicted HFRS occurrence ratios, we found that the accuracy of the predictions for the different land-use types differed between 2014 and 2015. In 2014, the accuracy of our predictions was higher for cultivated land, built-on land, and water bodies and lower for the other two types of land. In 2015, however, the accuracy of our predictions was lower for cultivated and forest and higher for the other three types of land. In both years, the predicted occurrence of HFRS in grassland was higher than the observed occurrence, while the predicted occurrence of HFRS in forest was relatively lower than the actual observation. Continuous pest control was carried out during the study period, which might have brought some unavoidable influences on the rodent community composition and even to the occurrence of HFRS. The prediction errors for water bodies were small because of the sustained small total number of HFRS cases on the type of land.

The influence of the rodent community composition on hantavirus transmission has been evaluated in previously studies [47,48]. We found that *M*. *musculus* and *R*. *norvegicus* were the predominant species in Chenzhou and both species played essential roles in hantavirus transmission, a finding similar to the situation for residential habitats in the Jiuhua Mountain area [47]. However, our results were more detailed in terms of the influence of the different rodent species on HFRS incidence. Nevertheless, the domestic and peridomestic habitats were found to be areas with more abundant reservoirs of hantavirus [48]. In the present study, cultivated land and built-on land, which are known to be closely related with human activities, were also found to be associated with higher risks of HFRS incidence. Moreover, the different rodent species were primarily affected by different environmental factors, which may indicate that once an environment changes, the rodent community composition may also change in response, thus influencing the transmission of hantavirus. However, some rodent species are habitat generalists, and if these species account for large parts of the overall rodent population, the infectious risk of HFRS would more likely to be determined by the infectious ability of the habitat generalist.

This study has some limitations. First, the prevalence of hantavirus in rodents was not considered because proper data for this was unavailable. Second, some factors may have caused inaccuracy in the division of land-use types for the HFRS cases: 1) the residential addresses were geo-located via Google Earth, which may have led to inaccurate land-use types via spatial resolution issues; 2) the validity of our findings depends on the assumptions that the location of the residence determines the risk of infection but the site of infection maybe not coincide completely with the residency of the individuals; in fact, they might be infected accidently by exposure far away from their residency. Third, due to the limitation of the rodent surveillance data, it is quite difficult to apply the analysis on monthly or seasonal basis. Fourth, the generalist species should be detected and taken into consideration in a further study. Finally, the sex and age structure of both the rodent populations and the susceptible populations were not considered in detail, but these factors might influence the transmission of hantavirus and the occurrence of HFRS.

### Conclusion

In this study, we assessed the distribution of rodent species across different land types and ascertained the primary species in regions with HFRS. Our findings can be used to support adjustments in rodent surveillance programs and to reduce the occurrence of HFRS in diverse environments. We also predicted the risk of HFRS for different land types in 2014 and 2015, and quantified the associations among the occurrence of HFRS, rodent species, and environmental factors by constructing and calculating matrices. These methods can be utilized to predict the risk of HFRS in the future, in Chenzhou and in other areas with similar characteristics. Our work has not only provided scientific data for improving HFRS prevention and control strategies but also serve as methodological advancement for use with similar zoonotic infections.

## Supporting information

S1 Table Annual number of reported HFRS cases among different land types during 2006–2015. (DOCX)

S2 Table The experience coefficients (a and b) in the TVDI calculation. (DOCX)

S3 Table Potential contact matrix *β*. (DOCX)

S4 Table Environmental coefficients matrix *α*. Tem: temperature, H: relative humidity, Pre: precipitation, NDVI: the normalized difference vegetation index, TVDI: the temperature vegetation dryness index.
(DOCX)

S1 Fig The land use types in Chenzhou City during 2006–2015. (TIF)
