# Supplementary material for: Spatial heterogeneity of hemorrhagic fever with renal syndrome is driven by environmental factors and rodent community composition
Source: PLoS Negl Trop Dis. 2018 Oct 24;12(10):e0006881. doi: 10.1371/journal.pntd.0006881 (PMC6218101; doi:10.1371/journal.pntd.0006881)
Supplement: S1 Table — (DOCX) [file pntd.0006881.s001.docx]

**S1 Table. The annual number of reported HFRS cases among different land types during 2006-2015.**

|  | 2006 | 2007 | 2008 | 2009 | 2010 | 2011 | 2012 | 2013 | 2014 | 2015 |
| --- | --- | --- | --- | --- | --- | --- | --- | --- | --- | --- |
| Cultivated land | 46 | 43 | 31 | 34 | 32 | 55 | 78 | 44 | 33 | 33 |
| Forest | 4 | 0 | 7 | 6 | 1 | 5 | 11 | 9 | 7 | 10 |
| Grass | 11 | 13 | 12 | 8 | 6 | 14 | 16 | 18 | 7 | 8 |
| Building land | 8 | 15 | 7 | 14 | 7 | 10 | 8 | 14 | 11 | 8 |
| Water body | 1 | 0 | 0 | 1 | 0 | 0 | 2 | 1 | 0 | 0 |
| Total | 70 | 71 | 57 | 63 | 46 | 84 | 115 | 86 | 58 | 59 |
