# Supplementary material for: Spatial heterogeneity of hemorrhagic fever with renal syndrome is driven by environmental factors and rodent community composition
Source: PLoS Negl Trop Dis. 2018 Oct 24;12(10):e0006881. doi: 10.1371/journal.pntd.0006881 (PMC6218101; doi:10.1371/journal.pntd.0006881)
Supplement: S2 Table — (DOCX) [file pntd.0006881.s002.docx]

**S2 Table. The experience coefficients (a and b) in the TVDI calculation.**

|  |  | Dry |  | Wet |  |
| --- | --- | --- | --- | --- | --- |
| Year | Month | a | b | a | b |
| 2006 | 1 | 286.8 | 1.11 | 285.98 | -7.57 |
| 2006 | 2 | 297.09 | -6.38 | 295.11 | -15.96 |
| 2006 | 3 | 301.08 | -5.78 | 297.22 | -8.88 |
| 2006 | 4 | 300.37 | -2.7 | 296.55 | -5.54 |
| 2006 | 5 | 305.39 | -2.08 | 302.23 | -11.91 |
| 2006 | 6 | 300.6 | 4.21 | 236.52 | -95.83 |
| 2006 | 7 | 307.99 | -5.16 | 261.04 | -59.59 |
| 2006 | 8 | 309.2 | -8.27 | 305.34 | -10.86 |
| 2006 | 9 | 302.96 | -3.18 | 306.06 | -13.87 |
| 2006 | 10 | 300.77 | -0.86 | 301.98 | -11.05 |
| 2006 | 11 | 300.12 | -2.69 | 301.84 | -14.8 |
| 2006 | 12 | 290.28 | -3.07 | 289.55 | -7.32 |
| 2007 | 1 | 289.08 | -5.02 | 288.2 | -10.8 |
| 2007 | 2 | 302.06 | -11.26 | 295.67 | -10.29 |
| 2007 | 3 | 298.16 | 1.21 | 318.15 | -84.9 |
| 2007 | 4 | 300.72 | -5.32 | 299.74 | -13.98 |
| 2007 | 5 | 305.84 | -3.73 | 304.45 | -10.3 |
| 2007 | 6 | 297.91 | 6.88 | 266.27 | -119.94 |
| 2007 | 7 | 259.88 | 60.18 | 28.33 | -37.45 |
| 2007 | 8 | 313.3 | -10.01 | 310.37 | -17.1 |
| 2007 | 9 | 307.41 | -8.11 | 308.44 | -16.86 |
| 2007 | 10 | 300.64 | -2.43 | 303.3 | -13 |
| 2007 | 11 | 297.03 | -4.48 | 297.25 | -11.4 |
| 2007 | 12 | 292.38 | -3.98 | -142.07 | 490.29 |
| 2008 | 1 | 294.74 | -6.8 | 288.56 | -9.98 |
| 2008 | 2 | 298.05 | -12.4 | 284.14 | -2.83 |
| 2008 | 3 | 300.83 | -6.82 | 294.42 | -4.47 |
| 2008 | 4 | 304.32 | -4.16 | 301.92 | -13.07 |
| 2008 | 5 | 304.47 | -3.15 | 304.66 | -13.15 |
| 2008 | 6 | 250.76 | 73005 | 212.12 | -90.05 |
| 2008 | 7 | 322.1 | -22.09 | 308.66 | -12.88 |
| 2008 | 8 | 315.88 | -14.9 | 297.12 | -7.64 |
| 2008 | 9 | 311.26 | -11.85 | 306.09 | -11.92 |
| 2008 | 10 | 307.95 | -11.01 | 307.12 | -16.67 |
| 2008 | 11 | 395.81 | -4.84 | 296.42 | -13.66 |
| 2008 | 12 | 296.14 | -7.93 | 294.15 | -12.29 |
| 2009 | 1 | 289.81 | -3.69 | 288.1 | -7.59 |
| 2009 | 2 | 307.04 | -15.39 | 298.11 | -9.89 |
| 2009 | 3 | 300.19 | -7.81 | 293.5 | -8.35 |
| 2009 | 4 | 302.94 | -5.66 | 301.75 | -13.69 |
| 2009 | 5 | 303.21 | -0.43 | 306.11 | -14.21 |
| 2009 | 6 | 307.05 | -3.62 | 308.75 | -14.42 |
| 2009 | 7 | 310.53 | -7.93 | 309.08 | -14.16 |
| 2009 | 8 | 315.18 | -14.12 | 306.8 | 11.91 |
| 2009 | 9 | 309.02 | -7.15 | 307.96 | -15 |
| 2009 | 10 | 305.24 | -6.08 | 304.31 | -12.97 |
| 2009 | 11 | 297.63 | -4.8 | 298.92 | -13.74 |
| 2009 | 12 | 292.8 | -8.36 | 290.92 | -13.7 |
| 2010 | 1 | 298 | -11.3 | 248 | 10.7 |
| 2010 | 2 | 304 | -11.4 | 188 | 156 |
| 2010 | 3 | 305 | -12.2 | 269 | -24.2 |
| 2010 | 4 | 303 | -4.93 | 244 | 25.3 |
| 2010 | 5 | 299 | 12.3 | 220 | 89.9 |
| 2010 | 6 | 307 | -2.96 | 203 | 106 |
| 2010 | 7 | 312 | -7.37 | 271 | 0.27 |
| 2010 | 8 | 315 | -6.4 | 290 | -27.6 |
| 2010 | 9 | 314 | -9.32 | 206 | 91.6 |
| 2010 | 10 | 304 | 5.46 | 353 | -116 |
| 2010 | 11 | 305 | -7.18 | 340 | -101 |
| 2010 | 12 | 296 | -6.36 | 294 | -9.36 |
| 2011 | 1 | 277 | -3.73 | 262 | -11.2 |
| 2011 | 2 | 285 | -3.84 | 263 | -4.48 |
| 2011 | 3 | 284 | -0.97 | 278 | -31.3 |
| 2011 | 4 | 293 | -0.91 | 355 | -149 |
| 2011 | 5 | 293 | 2.61 | 240 | 39.5 |
| 2011 | 6 | 300 | 1.39 | 294 | -20.4 |
| 2011 | 7 | 296 | 5.88 | 216 | 74.6 |
| 2011 | 8 | 300 | 1.12 | 335 | -73.1 |
| 2011 | 9 | 299 | -2.85 | 273 | 7.48 |
| 2011 | 10 | 293 | -1.41 | 272 | 0.53 |
| 2011 | 11 | 292 | -2.59 | 267 | 3.52 |
| 2011 | 12 | 283 | -3.44 | 290 | -55.9 |
| 2012 | 1 | 284.12 | 2.75 | 280.61 | -5.81 |
| 2012 | 2 | 289.23 | -0.75 | 286.78 | -4.5 |
| 2012 | 3 | 299.49 | -5.06 | 296.51 | -7.36 |
| 2012 | 4 | 308.049 | -9.6 | 304.88 | -15.61 |
| 2012 | 5 | 304.11 | 3.84 | 292.38 | 2.08 |
| 2012 | 6 | 306.35 | 1.16 | 294.28 | 3.25 |
| 2012 | 7 | 303.59 | 2.19 | 294.99 | 0.87 |
| 2012 | 8 | 305.87 | -4.15 | 292.75 | 3024 |
| 2012 | 9 | 305.11 | -7.19 | 294.59 | -5.03 |
| 2012 | 10 | 299.34 | -10.72 | 291.49 | -5.77 |
| 2012 | 11 | 288.03 | -9.29 | 256.32 | 42.01 |
| 2012 | 12 | 287.63 | -8.06 | 267.98 | 10.12 |
| 2013 | 1 | 289.35 | -1.1 | 283.2 | -4.22 |
| 2013 | 2 | 18 | 180 | NA | NA |
| 2013 | 3 | 306 | 0 | 290 | 0 |
| 2013 | 4 | 306 | 0 | 292 | 0 |
| 2013 | 5 | 312.61 | -4.27 | 299.3 | -4.04 |
| 2013 | 6 | 313.98 | -8.35 | 299.49 | -6.36 |
| 2013 | 7 | 311 | 0 | 292 | 0 |
| 2013 | 8 | 310.5 | -8.79 | 303.71 | -12.07 |
| 2013 | 9 | 302.07 | -3.15 | 295.16 | -0.95 |
| 2013 | 10 | 298.46 | -2.31 | 291.12 | -0.46 |
| 2013 | 11 | 294.38 | -7.33 | 287.81 | -8.43 |
| 2013 | 12 | 286.59 | -1.48 | 281.14 | -0.04 |
| 2014 | 1 | 288 | -2.96 | 284.94 | -6.18 |
| 2014 | 2 | 296.72 | -5.73 | 291.08 | -8.95 |
| 2014 | 3 | 302.62 | -6.39 | 299.89 | -11.48 |
| 2014 | 4 | 308.61 | -6.82 | 304.62 | -14.69 |
| 2014 | 5 | 306.74 | -7.24 | 286.82 | 17.93 |
| 2014 | 6 | 303.41 | 5.11 | 295.71 | -0.6 |
| 2014 | 7 | 307.13 | -2.39 | 296.95 | 1.17 |
| 2014 | 8 | 305.34 | -5.27 | 291.48 | 3.64 |
| 2014 | 9 | 304.95 | -6.96 | 295.29 | -5.19 |
| 2014 | 10 | 298.93 | -4.57 | 293.61 | -8.9 |
| 2014 | 11 | 299.99 | -7.97 | 267.41 | 16.99 |
| 2014 | 12 | 291.24 | -3.44 | 286.49 | -4.54 |
| 2015 | 1 | 287.55 | -2.79 | 284.03 | -8.89 |
| 2015 | 2 | 300.69 | -6.79 | 245.96 | 46.21 |
| 2015 | 3 | 303.6 | -6.38 | 297.72 | -8.24 |
| 2015 | 4 | 307.64 | -8.79 | 303.01 | -12.2 |
| 2015 | 5 | 303.68 | 4.02 | 293.74 | 3.72 |
| 2015 | 6 | 305.49 | 2.66 | 297.55 | -1.15 |
| 2015 | 7 | 307.05 | -2.07 | 294.41 | 0.02 |
| 2015 | 8 | 306.3 | -5.83 | 301.39 | -0.56 |
| 2015 | 9 | 303.84 | -5.36 | 292.67 | -6.33 |
| 2015 | 10 | 302.43 | 0.37 | 270.88 | 23.54 |
| 2015 | 11 | 292.94 | -4.39 | 281.16 | 0.36 |
| 2015 | 12 | 290.77 | -4.19 | 283.21 | -2.21 |
