# Supplementary material for: Spatial heterogeneity of hemorrhagic fever with renal syndrome is driven by environmental factors and rodent community composition
Source: PLoS Negl Trop Dis. 2018 Oct 24;12(10):e0006881. doi: 10.1371/journal.pntd.0006881 (PMC6218101; doi:10.1371/journal.pntd.0006881)
Supplement: S3 Table — (DOCX) [file pntd.0006881.s003.docx]

**S2 Table. The potential contact matrix *β*.**

|  | *R. norvegicus* | *M. musculus* | *R. flavipectus* | Others |
| --- | --- | --- | --- | --- |
| Cultivated land | 0.507671 | 0.661923 | 0.62064 | 1.70278 |
| Forest | 0.157001 | 0.078147 | -0.57226 | 1.92659 |
| Grassland | 0.333159 | 0.037906 | 0.333294 | -1.26579 |
| Building land | -0.01307 | 0.213218 | 0.712423 | -1.84608 |
| Water body | 0.015242 | 0.008806 | -0.0941 | 0.482498 |
