# Supplementary material for: Spatial heterogeneity of hemorrhagic fever with renal syndrome is driven by environmental factors and rodent community composition
Source: PLoS Negl Trop Dis. 2018 Oct 24;12(10):e0006881. doi: 10.1371/journal.pntd.0006881 (PMC6218101; doi:10.1371/journal.pntd.0006881)
Supplement: S4 Table — Tem: temperature, H: relative humidity, Pre: precipitation, NDVI: the normalized difference vegetation index, TVDI: the temperature vegetation dryness index. (DOCX) [file pntd.0006881.s004.docx]

**S3 Table. The environmental coefficients matrix *α*.** Tem: temperature, H: relative humidity, Pre: precipitation, NDVI: the normalized difference vegetation index, TVDI: the temperature vegetation dryness index.

|  | *R. norvegicus* | *M. musculus* | *R. flavipectus* | Others |
| --- | --- | --- | --- | --- |
| Tem | 0.279207 | 1.917787 | -0.44602 | -0.15772 |
| H | 1.895108 | 6.214905 | -5.07008 | -2.03153 |
| Pre | 0.014556 | -0.48892 | 0.547457 | 0.180416 |
| NDVI | -0.06342 | -1.51911 | 1.000468 | 0.553746 |
| TVDI | 0.324686 | -0.64522 | 0.323203 | 0.013622 |
