# Supplementary figures and images for: Spatial heterogeneity of hemorrhagic fever with renal syndrome is driven by environmental factors and rodent community composition
Source: PLoS Negl Trop Dis. 2018 Oct 24;12(10):e0006881. doi: 10.1371/journal.pntd.0006881 (PMC6218101; doi:10.1371/journal.pntd.0006881)

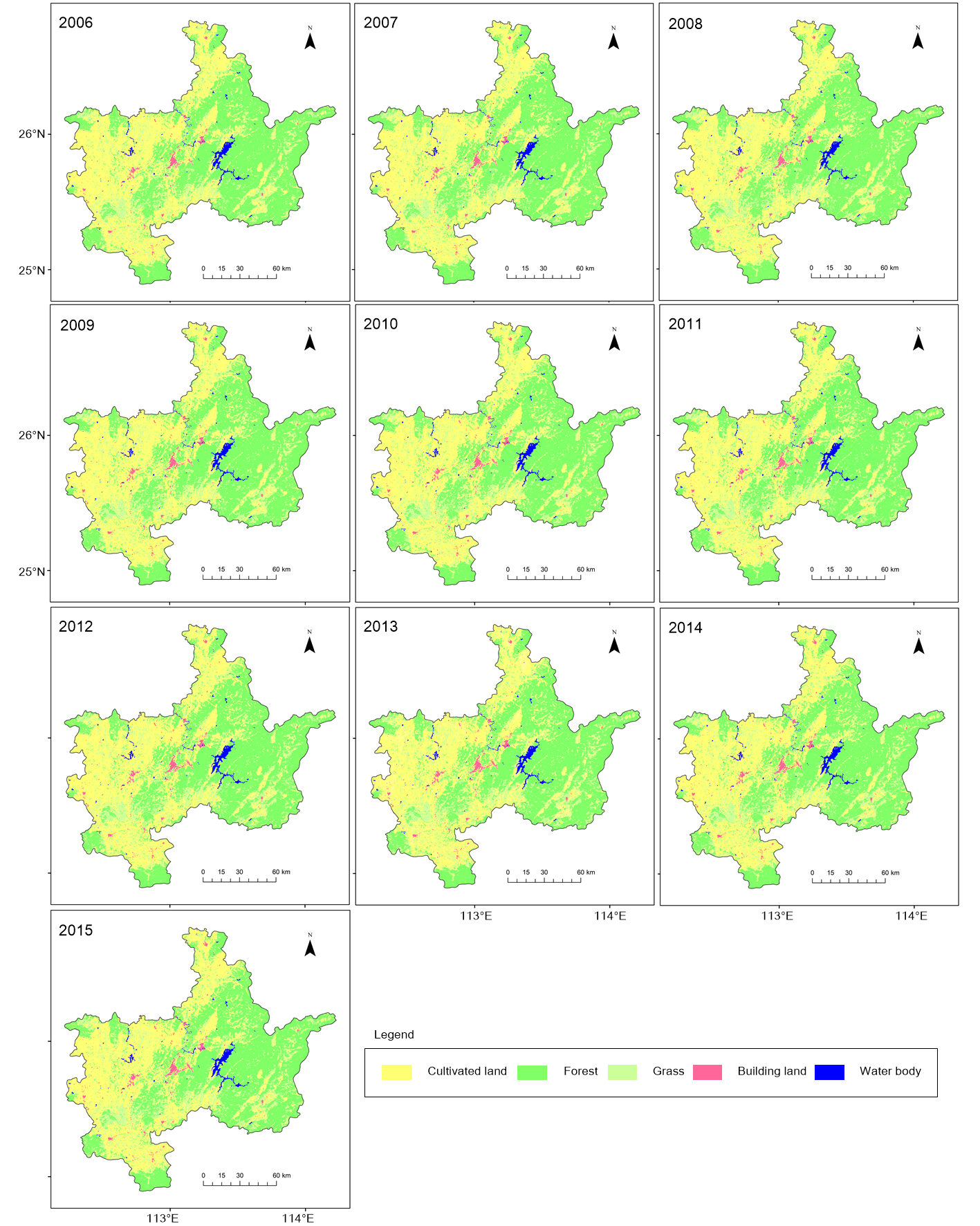

Supplement: S1 Fig — (TIF) [file pntd.0006881.s005.tif]
